# Measurement of pH micro-heterogeneity in natural cheese matrices by fluorescence lifetime imaging

**DOI:** 10.3389/fmicb.2015.00183

**Published:** 2015-03-06

**Authors:** Zuzana Burdikova, Zdenek Svindrych, Jan Pala, Cian D. Hickey, Martin G. Wilkinson, Jiri Panek, Mark A. E. Auty, Ammasi Periasamy, Jeremiah J. Sheehan

**Affiliations:** ^1^Teagasc Food Research CentreMoorepark, Fermoy, Ireland; ^2^Department of Biology, W. M. Keck Center for Cellular Imaging, University of VirginiaCharlottesville, VA, USA; ^3^Department of Sport Medicine, Third Faculty of Medicine, Charles UniversityPrague, Czech Republic; ^4^Department of Life Sciences, Faculty of Science and Engineering, University of LimerickLimerick, Ireland; ^5^Institute of Macromolecular Chemistry, Academy of Sciences of the Czech RepublicPrague, Czech Republic

**Keywords:** cheese matrix, C-SNARF-4, Oregon Green, pH micro-heterogeneity, fluorescence lifetime, FLIM, natural cheese

## Abstract

Cheese, a product of microbial fermentation may be defined as a protein matrix entrapping fat, moisture, minerals and solutes as well as dispersed bacterial colonies. The growth and physiology of bacterial cells in these colonies may be influenced by the microenvironment around the colony, or alternatively the cells within the colony may modify the microenvironment (e.g., pH, redox potential) due to their metabolic activity. While cheese pH may be measured at macro level there remains a significant knowledge gap relating to the degree of micro-heterogeneity of pH within the cheese matrix and its relationship with microbial, enzymatic and physiochemical parameters and ultimately with cheese quality, consistency and ripening patterns. The pH of cheese samples was monitored both at macroscopic scale and at microscopic scale, using a non-destructive microscopic technique employing C-SNARF-4 and Oregon Green 488 fluorescent probes. The objectives of this work were to evaluate the suitability of these dyes for microscale pH measurements in natural cheese matrices and to enhance the sensitivity and extend the useful pH range of these probes using fluorescence lifetime imaging (FLIM). In particular, fluorescence lifetime of Oregon Green 488 proved to be sensitive probe to map pH micro heterogeneity within cheese matrices. Good agreement was observed between macroscopic scale pH measurement by FLIM and by traditional pH methods, but in addition considerable localized microheterogeneity in pH was evident within the curd matrix with pH range between 4.0 and 5.5. This technique provides significant potential to further investigate the relationship between cheese matrix physico-chemistry and bacterial metabolism during cheese manufacture and ripening.

## Introduction

Cheese is a complex physiochemical and microbial system containing many interacting components. It may be defined as a protein matrix entrapping fat, moisture, minerals and other solutes as well as dispersed bacterial colonies. Irrespective of cheese type, both starter and non-starter bacteria are immobilized and isolated within the curd matrix during the coagulation step of cheese manufacture (Fox et al., 2000; McSweeney, 2004b; Jeanson et al., 2011). Each inoculated bacterial cell is assumed to grow, generating colonies which are dispersed within the cheese curd. Bacterial colonies within the cheese matrix have been shown to be located on or within milk fat globular membrane material (MFGM) and at fat-protein interfaces with the curd (Laloy et al., 1996; Lopez et al., 2006) and are considered to interact with the cheese matrix during ripening (Feeney et al., 2002; McSweeney, 2004a). The growth and physiology of bacterial cells in these colonies may be influenced by the microenvironment around the colony, or alternatively the cells within the colony may modify the microenvironment (e.g., pH, redox potential) due to their metabolic activity (McSweeney, 2004a).

The pH of cheese curd is determined by the extent of acidification during manufacture, by the availability of substrate for fermentation, principally lactose, by the buffering capacity of the cheese curd and, in some cases, by the degree of deacidification during ripening. Cheese pH affects the texture of curd directly by influencing the degree of casein hydration, which in turn influences the visco-elastic behavior of the protein matrix (Euston et al., 2002; Kilcast and Angus, 2007). pH also affects texture and flavor indirectly by influencing the activity of enzymes important to ripening, e.g., plasmin (Grufferty and Fox, 1988) and, in the case of the coagulant, both the retention of and the activity of the enzyme in the curd during manufacture and subsequent ripening (Holmes et al., 1977; Stadhouders et al., 1977; Visser, 1977; Creamer et al., 1985; Garnot et al., 1987). The pH also influences the metabolic activity of lactic acid bacteria (Meldrum et al., 2003; Kajfasz and Quivey, 2011; Jeanson et al., 2013). The pH is a key factor influencing amino acid decarboxylase activity (Gardini et al., 2001) and bacteriocin production (Foulquié Moreno et al., 2003).

Development of a method capable of precisely measuring pH at localized level within the cheese matrix will facilitate a new understanding of the relationship between cheese matrix physico-chemistry and bacterial metabolism during cheese manufacture and ripening.

Until now different techniques have been used to monitor pH in and around colonies. Microelectrodes were first used to measure pH in and around submerged colonies of *S. typhimurium* (Wimpenny et al., 1995; Jeanson et al., 2013), however, these cannot map the spatial variation of pH to a resolution of a micrometer. Standard methodologies to measure the pH in cell biology include the use of pH sensitive fluorescent dyes—fluorescein based pH indicator, benzoxanthene dyes, cyanine-based pH indicators, etc. (Han and Burgess, 2010). In the vast majority of these experiments Confocal Laser Scanning Fluorescence Microscopy (CLSM) is employed, mainly due to its ability to perform optical sectioning and provide accurate three-dimensional representation of the fluorophore distribution (Inoue, 1995).

Seminaphthorhodafluor-4F 5-(and-6) carboxylic acid (C-SNARF-4) is a long wavelength fluorescent pH indicator (Haugland, 2002). The dye exhibits two emission peaks whose intensities display different pH dependencies, thus it is suitable as a ratiometric fluorescent pH probe. The results of the fluorescence intensity ratio of these two peaks have been confirmed as a reliable indicator of pH at values 5.0 and above (Hunter and Beveridge, 2005). However, in more acidic environments the pH sensitivity of the fluorescence ratio decreases rapidly.

Oregon Green 488 is a fluorinated analog of fluorescein. It exhibits higher photostability and lower pK_a_ (pK_a_ = 4.7 vs. 6.4 for fluorescein), making it a useful pH indicator in the weakly acidic range (pH 4–6). However, to use Oregon Green as a ratiometric pH probe, two excitation wavelengths are necessary (Whitaker et al., 1991). Oregon Green carboxylic acid has been widely employed to measure lysosomal and endosomal pH (Dunn et al., 1991). Bioconjugate prepared from Oregon Green 488, dextran, has the advantage of high stability and low affinity to cheese matrix components (proteins).

Besides fluorescence intensity, fluorescence lifetime, a probabilistic timescale of fluorescence emission (Sun et al., 2011), may provide additional information about the chemical environment (e.g., pH) of the probe. The most common example is fluorescein, whose fluorescence lifetime changes from 4 ns at pH 10 to 2 ns at pH 7 (Lakowicz, 2006), and many other fluorophores were probed for lifetime–pH dependence, including some of the SNARF family (Szmacinski and Lakowicz, 1993). Oregon Green dyes were also probed for lifetime–Ca^2+^ sensitivity (Agronskaia et al., 2004).

### Fluorescence lifetime imaging (FLIM)

Fluorescence of a fluorophore is characterized by its absorption spectrum (a probability that a photon of given wavelength is absorbed by the molecule), quantum yield (a probability that the excited molecule emits a photon during transition to the ground state), its emission spectrum (a distribution of wavelengths of the emitted photon) and the fluorescence lifetime (a characteristic time the molecule spends in the excited state before emitting a photon). Both excitation and emission spectrum are used extensively to distinguish different fluorophores and to sense the local environment of a fluorophore (local ion concentration, electric potential, pH, etc., Lakowicz, 2006). However, only a limited number of fluorophores, often specifically designed, display useful changes in excitation and emission spectra. Here we note that changes in fluorescence intensity alone cannot be exploited in most cases, because the fluorescence intensity depends predominantly on the concentration of the fluorescent molecules. When such fluorophores are not available or do not perform optimally in specific experimental conditions, one may explore the dependence of fluorescence lifetime on the local environment.

**Figure 1:**
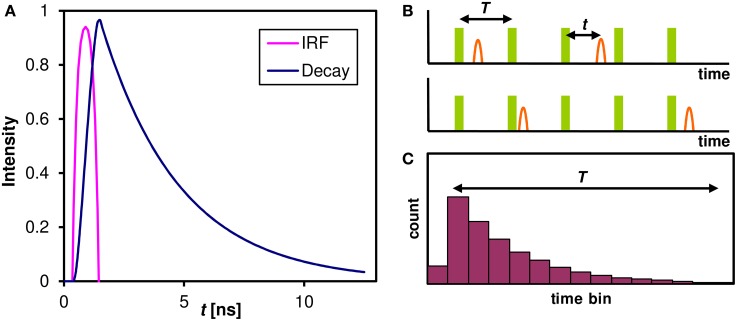
**The Fluorescence decay and its measurement. (A)** The fluorescence decay of an ensemble of identical molecules after a laser pulse. The Instrumental Response Function (IRF) combines the excitation pulse duration and the time resolution of the detector and an associated hardware. The intensity of a single fluorescent species that can only assume single ground and excited state follows an exponential decay with a characteristic lifetime τ. **(B)** In a practical TCSPC experiment there is typically much less than one photon detected for every laser pulse. The time of photon arrival *t* within the laser pulse period *T* is measured for every photon. In our case of 80 MHz laser pulse repetition rate the period *T* = 12.5 ns. **(C)** A histogram of photon arrival times that is built from the single photon events approaches the theoretical ensemble decay curve of **(A)**. This histogram can be built in each pixel of a confocal image and the lifetime information at each pixel is then extracted by fitting an exponential model (Equation 1).

The fluorescence lifetime has only recently been widely adopted, mainly because of the technical challenges associated with the measurement of such fast phenomena (typically in the nanosecond range). One of the most efficient precise and reliable methods used in conjunction with confocal microscopy is Time Correlated Single Photon Counting (TCSPC). This method relies on sub-nanosecond pulsed illumination and fast photon-counting detectors to measure the delay between the excitation pulse and the photon detection event (Becker, 2005). The intensity of fluorescence following a short excitation pulse (usually much shorter than 1 ns) decays exponentially on a nanosecond timescale (Figure 1A). This decay, however, cannot be digitized directly with current instrumentation; moreover, in a typical confocal microscopy experiment there is less than one photon detected in a hundred of laser pulses. Instead, the time delay between the laser pulse and photon detection event is measured (Figure 1B) for each photon and a histogram (Figure 1C) is built from these delays in each pixel of a confocal image. By fitting an exponential model (Equation 1) to these decays, the information about the lifetime of the constituents is recovered.

In simple cases (single molecular species with single ground and excited state configuration, or with configurations changing in sub-nanosecond timescale) single exponential decay completely describes the experimental data. However, often more exponential components are needed to achieve acceptable fit between the model (Equation 1) and the recorded decay. Although the detailed mechanism is often poorly understood, multi-exponential decay suggest the existence of more distinct fluorescent species or several stable configurations of the fluorophore on long timescales (relatively to the fluorescence lifetime). Despite the inherent difficulties and numerical instability of multi-exponential fitting, when performed cautiously, the individual lifetime components can provide valuable information regarding local environment of the fluorophore (Becker, 2005).

The objectives of this study were (i) to assess the potential of C-SNARF-4 and Oregon Green 488 fluorescent dyes to perform as ratiometric pH indicators in cheese matrices at microscale, (ii) to extend the useful pH range of the probes using lifetime measurements, and (iii) to determine the level of pH microheterogeneity within a natural cheese matrix. To our knowledge it is the first time that advanced microscopic technique fluorescent lifetime imaging microscopy (FLIM) of C-SNARF-4 and Oregon Green 488 has been applied to determine localized pH of different types of natural cheese.

## Materials and methods

### Cheese preparation and characterization

#### Starter strains

Thermophilic starter cultures typically used in Swiss-type cheese manufacture (Scott, 1981), i.e., *S. thermophilus* TH3 (ST) and *L. helveticus* LHB02 (LH) were purchased from Chr. Hansen Ltd. (Little Island, Co. Cork, Ireland) as individual frozen concentrates and stored at −80°C until cheese manufacture.

#### Cheese manufacture

Two cheese making trials consisting of four vats of 454 kg cheese milk were undertaken over a 6 month period. Raw milk was obtained from a local dairy company, standardized to a protein to fat ratio of 1.01:1, held overnight at 4°C, pasteurized at 72°C for 15 s, and pumped at 32°C into cylindrical, jacketed, stainless steel vats (500·l) with automated variable speed cutting and stirring equipment (APV) Schweiz AG, Worb, Switzerland). Cheesemilk was heated to 34°C and inoculated with 0.1 g/l ST (10^6^ cfu/ml) and 0.05 g/l LH (5·10^5^ cfu/ml). After a 60 min ripening period, chymosin (Chymax plus, Chr. Hansens Ltd.), diluted 1:6 with de-ionized water, was added at a level of 18 ml per 100 kg milk. A coagulation time of 35 min was allowed prior to the cutting of the coagulum. After a 10 min healing period, the curd/whey mixture was cooked by steam injection into the jacket of the vat with constant stirring. Maximum scalds employed were 50 or 40°C (Table 1). Curds were cooked at a rate of 0.5°C/min from 34 to 45°C and at 1°C/min from 45°C to maximum scald (50°C) where appropriate. Curds were pitched at pH 6.3.

**Table 1:** **Manufacture parameters used during cheese making trials**.

**Treatment**	**50°C, BS**	**50°C, DS**	**40°C, BS**	**40°C, DS**
Starter cultures	*S. thermophilus*	*S. thermophilus*	*S. thermophilus*	*S. thermophilus*
	*L. helveticus*	*L. helveticus*	*L. helveticus*	*L. helveticus*
Max scald	50°C	50°C	40°C	40°C
Curd handling	Pre-press and mold	Cheddaring of curd	Pre-press and mold	Cheddaring of curd
Salting method	Brine	Dry salt at 1.45% (w/w)	Brine	Dry salt at 1.45% (w/w)

Brine salted (BS) cheeses were pre-pressed under whey at 5.4 kPa for 10 min after which the curd was placed in 10 kg round molds and pressed under increasing pressure up to 35.2 kPa. At pH 5.3, the cheeses were de-molded and placed for 21 h in a saturated brine solution [23% (w/w) NaCl, 0.2% (w/w) Ca, pH 5.2, 10°C].

Dry salted (DS) cheeses were drained and the curd cheddared, milled at pH 5.3, salted at a rate of 1.45% (w/w) and pressed overnight on a horizontal press at 264.6 kPa in 20 kg blocks. All cheeses were vacuum packed post brine/pressing and ripened at 8°C for up to 230 days.

#### Macroscopic pH measurement

A standard procedure to measure the pH of cheese (BS 770-5:1976) was used. Briefly, a slurry was created from a cheese sample using distilled water at 20°C and the pH of the cheese suspension was recorded with a standard laboratory electrode-based pH meter. All samples were measured in duplicate and the mean values are provided.

### Fluorescence microscopy

#### Sample preparation

C-SNARF-4 calibration samples were prepared by mixing 10 μl of 1 mM C-SNARF-4 in DMSO stock solution with 90 μl of 100 mM sodium citrate buffer adjusted to pH 3.0–7.0. A 20 μl drop of the solution was placed on a glass coverslip and imaged with an inverted confocal microscope.

C-SNARF-4 stained cheese samples were prepared by cutting a small piece (about 5 × 5× 0.5 mm) from the bulk of the cheese, the piece was then placed on a glass coverslip with a 20 μl drop of fluorophore solution (10 μl of 1 mM C-SNARF-4 in DMSO stock solution mixed with 90 μl of deionized water). Additionally, cheese samples without the fluorescent probe were prepared to assess the amount of cheese autofluorescence.

Oregon Green 488 (Molecular Probes, D-7172) stained calibration samples were prepared by immersing a small piece of cheese in a 40 μl drop of Oregon Green 488 buffered solution (20 μl of 50 μM dye in deionized water, mixed with 20 μl of 100 mM sodium citrate buffer adjusted to pH 3.0–6.0) on a glass coverslip and imaged with an inverted microscope.

For cheese pH measurements the same protocol was followed, but the citrate buffer was replaced by an equal amount of deionized water to maintain the same dye concentration as in the calibration samples. To assess the cheese autofluorescence, cheese samples without the fluorescent probe were also prepared.

#### Fluorescence image acquisition

Spectrally resolved fluorescence measurements and excitation-emission spectra measurements of C-SNARF-4 were performed with Leica TCS SP8 X confocal laser scanning microscope based on Leica DMI6000 inverted fluorescent microscope (Leica Microsystems CMS GmbH) equipped with White Light Laser (freely tunable excitation in the spectral range 470–670 nm), acousto-optical beam splitter and hybrid detectors with a LightGate option. The Acousto-Optical Beam Modulator (AOBM) was set in the mode of constant output power over the whole working interval that allows the constant intensity of the excitation laser beam for all excitation wavelengths impinging on the sample. The emission spectra were collected sequentially with internal spectral detector, with spectral bandwidth of 20 nm and step size 20 nm in the range 500–720 nm with the starting offset 10 nm from the excitation wavelength. We did not take into account cross-excitation and possible fluorescence emitted in the range less than 10 nm from the excitation wavelength. The image acquisition and analysis were performed in software LAS AF 3.1.2 (Leica Microsystems GmbH). Data were averaged from 200 × 200 μm area, approximately 30 μm deep in the calibration solution with a 20×/0.75 immersion objective lens.

#### Fluorescence lifetime measurements

Lifetime measurements of C-SNARF-4 calibration samples and C-SNARF-4 stained cheese samples were performed with Olympus FV1200 confocal laser scanning microscope based on Olympus IX83 inverted fluorescence microscope (Olympus Europe GmbH) equipped with two single photon counting hybrid detectors, two channel time correlator and 485 nm pulsed picosecond laser (PicoQuant GmbH). The laser repetition rate was set to 40 MHz and the laser power was adjusted to keep the mean detected photon rate below 2% of the laser repetition rate. Two spectral ranges were measured simultaneously (channel 1 corresponding to 570–620 nm, channel 2 corresponding to 650–700 nm). Typically, calibration data were averaged from 100 × 100 μm area, approximately 30 μm deep in the calibration solution with a 60×/1.2 water immersion objective lens. Typically, 120 s acquisition was necessary to accumulate 10^5^ detection events. When measuring cheese samples the focus was set to the cheese surface, i.e., the plane of the strongest fluorescence. The measured fluorescence decay curves were fitted to a two- or three-component exponential model (a sum of two or three exponential decays) according to

(1) $$I(t)=\sum_{n\;=\;1}^{N}a_{n}e^{-t/\tau_{n}}+b$$

using the PicoQuant SymPhoTime64 TCSPC fitting software. Here *I*(*t*) is the model fluorescence intensity, *N* is the number of components of the model, *I_n_* and τ*_n_* are the intensity and the lifetime of the *n*-th component of the model respectively (*n* = 1, 2, 3; lifetimes τ*_n_* arranged in ascending order) and *b* is the mean background. The fluorescence images were acquired at 512 × 512 pixels that were binned to 256 × 256 pixels for lifetime calculations.

Lifetime measurements of Oregon Green 488 stained cheese samples were performed with Zeiss LSM780 NLO confocal microscope based on Axio Observer. Z1 inverted microscope (Carl Zeiss MicroImaging GmbH) equipped with Chameleon Vision femtosecond pulsed IR laser (Coherent Inc.) tuned to 860 nm and Becker and Hickl hybrid detector (HPM-100–40) and TCSPC hardware and software (SPC-150, Becker & Hickl GmbH). The repetition rate of the pulsed laser was fixed to 80 MHz. Fluorescence was collected through an IR blocking filter and 580–640 nm emission filter with a Plan Apo 63×/1.4 oil IR objective lens. Fluorescence lifetimes were calculated from 512 × 512 pixel images binned to 256 × 256 pixels. The measured fluorescence decay curves were analyzed in terms of two-exponential model according to Equation (1) and the mean lifetime was then calculated according to

(2) $$\tau_{m}=\frac{\sum_{n\;=\;1}^{N}a_{n}\tau_{n}}{\sum_{n\;=\;1}^{N}a_{n}}$$

using the Beker & Hickl SPCImage software. Here τ*_m_* is the mean lifetime, other symbols are defined in Equation (1).

## Results

### Macroscopic pH of natural cheese samples

The results obtained through the standard measurement with a pH electrode of homogenized slurry samples are summarized in Table 2. It is evident that the cheese pHs vary depending on the cheese manufacture treatments but all are within the range 4.99–5.31 and are typical of the pH of many natural cheese types However, what this method does not measure is the degree and pattern of variability in pH within each cheese matrix. It is possible to get approximate measurement by direct measurement with a pH electrode of points within the cheese matrix but this is not sufficiently accurate to create a full pH map of the matrix and to relate the pH map to localized compositional, biochemical and microbial differences within the cheese matrix.

**Table 2:** **Macroscopic pH values of cheese trials at day 223 of ripening**.

**Sample**	**vat 1**	**vat 2**	**vat 3**	**vat 4**
Treatment	50°C, BS	50°C, DS	40°C, BS	40°C, DS
pH	5.20 ± 0.01	5.31 ± 0.01	4.99 ± 0.01	5.09 ± 0.01

### C-SNARF-4 emission spectra

**Figure 2:**
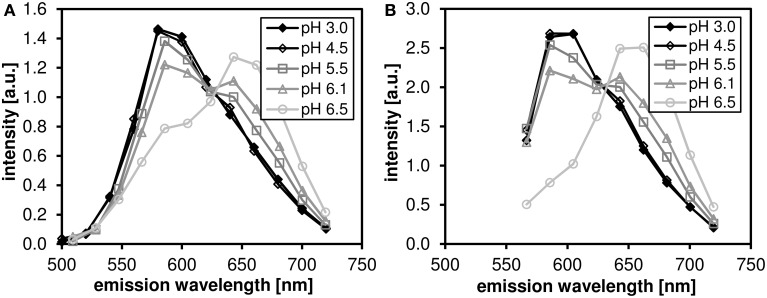
**The pH-dependent emission spectra of C-SNARF-4 in water**. Measured in the pH range 3.0–6.5 at two excitation wavelengths: **(A)** 488 nm, **(B)** 530 nm. Note that the spectra do not change significantly below pH 4.5.

**Figure 3:**
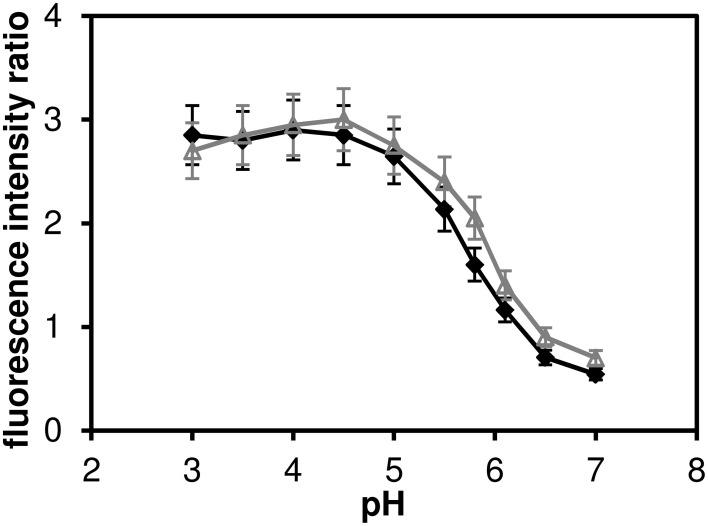
**The ratio of fluorescence intensities *I*_590_*I*_660_ of C-SNARF-4 ratiometric pH probe in water**. Measured in the pH range of 3.0–7.0, excited at 480 nm (solid symbols) ad at 530 nm (open symbols). The widths of the two emission bands (centered at 590 and 660 nm, respectively) are 60 nm.

The pH dependence of the probe's emission spectrum is summarized in Figure 2 for two excitation wavelengths (480 nm—the wavelength used for subsequent lifetime measurements; and 530 nm—the wavelength of most efficient excitation) in the pH range 3.0–7.0. The pH dependence of the ratio of the intensities of the two emission peaks (centered at 590 and 660 nm, respectively) is shown in Figure 3.

### C-SNARF-4 excitation-emission spectra

**Figure 4:**
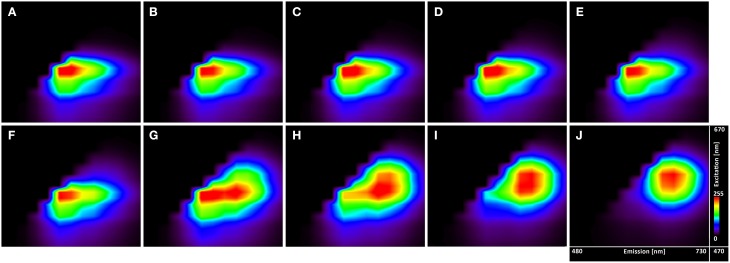
**Excitation-emission lambda scans of the C-SNARF-4 in water buffered to different pH**. Vertical axis shows excitation wavelengths of White Light Laser (470–670 nm), whereas horizontal axis displays emission spectra (480–730 nm) of C-SNARF-4 in different pH: **(A)** 3.7, **(B)** 4.0, **(C)** 4.3, **(D)** 4.5, **(E)** 4.8, **(F)** 5.0, **(G)** 5.5, **(H)** 5.8, **(I)** 6.1, and **(J)** 6.5. Increase of pH value results in moving maximum emission from the blue part of spectra toward the red part of spectra with forming two peaks horizontally for pH 5.5 and quasi-symmetrical emission for pH 6.1 and 6.5.

**Figure 5:**
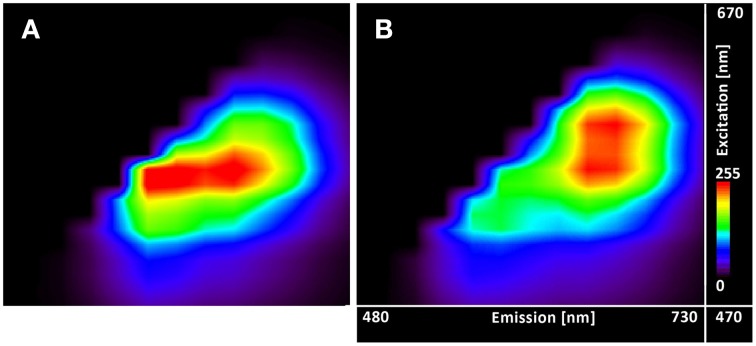
**Excitation-emission lambda scans of the C-SNARF-4 in water**. Measured at pH 5.5 **(A)** without timegate and **(B)** with timegate 1 ns, i.e., all photons arriving within the first nanosecond after the excitation pulse are discarded. Vertical axis shows excitation wavelengths of White Light Laser (470–670 nm), horizontal axis displays emission spectra (480–730 nm).

The excitation-emission scans (Figure 4) indicate almost no pH sensitivity of the dye in the pH range of 3.7–5.0 (the Figures 4A–F show the same spectral footprint regardless of pH). At pH 5 and above (Figures 4F–J) there is an apparent trend in the spectra that manifests the pH sensitivity of the probe. However, introducing time gating (discarding photons that arrive within 1 ns of the excitation pulse) modifies the spectral footprint of the (Figure 5) suggesting that lifetime imaging (FLIM) may enhance pH sensitivity at lower pH.

### C-SNARF-4 fluorescence lifetimes in aqueous solution

We performed calibration measurements of C-SNARF-4 dye in aqueous pH buffers in the range of pH 3.0–7.0 and measured the fluorescence lifetimes in two spectral bands: channel 1 corresponds to 570–620 nm, and channel 2 corresponds to 650–700 nm. These two spectral bands were chosen according to the two emission peaks of the dye used for ratiometric pH imaging.

**Figure 6:**
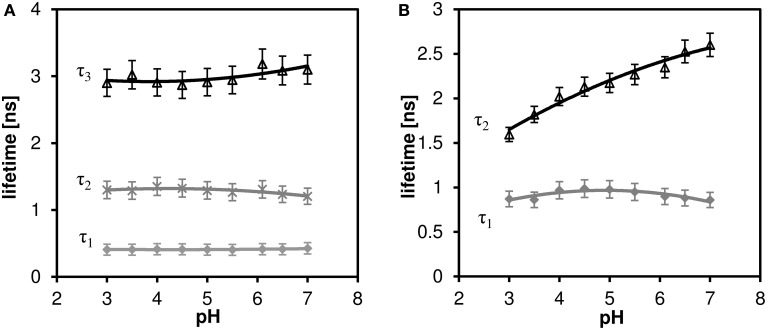
**The effect of pH on the fluorescence lifetime of C-SNARF-4 in water. (A)** Channel 1 (570–620 nm emission), three-component model, none of the lifetime components shows pronounced dependence. **(B)** Channel 2 (650–700 nm emission), two-component model, the longer lifetime component τ_*2*_ shows pronounced pH dependence.

While a two-exponential model fits sufficiently well the decay curves of channel 2 (far red fluorescence), three-exponential model is necessary for analyzing channel 1 (orange fluorescence). The results of the multi-exponential fitting procedure, i.e., pH dependence of the individual lifetime components are shown in Figure 6A (channel 1) and Figure 6B (channel 2). While the microscopic origin of the individual components is unknown, they can be used empirically to measure local pH, as long as they display sufficient sensitivity (the lifetime changes monotonically with pH and this change is bigger than the statistical error of the measurement). None of the channel 1 lifetime components displayed pronounced sensitivity toward pH. On the other hand, the long lifetime component of the channel 2 decay curves shows pronounced pH dependence in the whole measured pH range (the upper curve in Figure 6B). Mean fluorescence lifetime τ*_m_* calculated according to Equation (2) does not show pronounced pH dependence, thus two-exponential fitting is necessary to extract the pH information.

### Microscopic pH of cheese samples measured with C-SNARF-4

Based on calibration data we tried to utilize the pH dependence of C-SNARF-4 fluorescence in the red spectral channel for cheese pH measurements on the microscopic scale. However, both the fluorescence intensity and the fluorescence lifetime distribution were essentially homogeneous across the sample. We have also observed a strong affinity of the dye toward the cheese surface.

We measured a total of 4 cheeses (designated vat 1 to vat 4), each multiple times. Surprisingly, a new decay component with relatively long lifetime (τ_3_ ≥ 3.5 ns) appeared (Table 3). Moreover, the τ_2_ component, which is assumed to correspond to the pH dependent component of the calibration data, has significantly shorter lifetime than the value deduced from calibration (expected value is about 2.2 ns for pH 4.5). Also the fastest component (τ_1_ ≈ 0.7 ns) is significantly shorter, than the corresponding lifetime from calibration (about 0.9 ns). Thus, in our scenario the C-SNARF-4 probe did not prove itself as a useful probe of the local pH.

**Table 3:** **The fluorescence lifetimes of cheese matrix stained with C-SNARF-4 fluorescent dye, channel 2, three-component exponential model**.

	**τ_1_[ns]**	**τ_2_[ns]**	**τ_3_[ns]**
vat 1	0.62 ± 0.05	1.65 ± 0.09	3.71 ± 0.18
vat 2	0.61 ± 0.02	1.63 ± 0.03	3.69 ± 0.16
vat 3	0.56 ± 0.01	1.59 ± 0.02	3.65 ± 0.08
vat 4	0.59 ± 0.03	1.60 ± 0.04	3.62 ± 0.09

*Average lifetime ± standard deviation calculated from 6 to 8 measurements*.

Control experiments performed on cheese samples without fluorescent probe revealed, that the cheese autofluorescence is almost two orders of magnitude weaker than C-SNARF-4 fluorescence, and its lifetime is significantly longer (over 6 ns) than that of the fluorescent probe, so that the autofluorescence does not interfere with our measurements.

### Oregon green 488 fluorescence lifetimes and microscopic pH measurements

**Figure 7:**
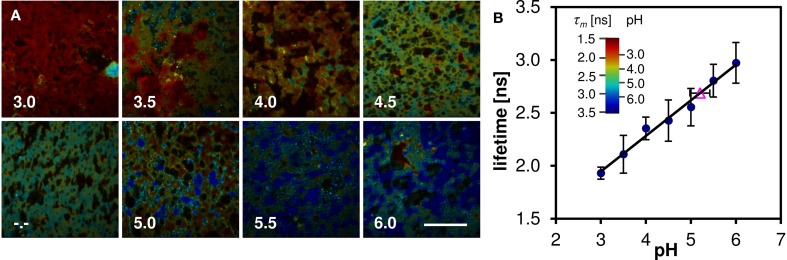
**Lifetime measurements of cheese stained with Oregon Green 488 buffered to pH 3.0–6.0. (A)** FLIM images, the pseudocolor represents the mean lifetime τ*_m_*, intensity represents photon count, captions denote the pH of the buffer solution (**-.-** denotes unbuffered sample). Scalebar 50 μm. **(B)** The pH dependence of mean lifetime τ*_m_* calculated from the FLIM images (solid symbols), and the mean lifetime of stained cheese without pH buffer (open triangle). Error bars represent the standard deviation, mainly caused by sample inhomogeneity. The inset shows the pseudocolor scale of the FLIM images calibrated both in lifetime and pH values.

Oregon Green 488 displays single emission peak whose position is independent of local environment. Thus fluorescence lifetime (FLIM) must be employed in order to measure the local pH variation. The mean fluorescence lifetime of the Oregon Green 488 excited by 860 nm pulsed laser at 80 MHz and detected in the red emission band was determined to be 3.9 ± 0.1 ns in aqueous buffer at pH 7. When the dye is applied to the cheese matrix, the lifetime shortens significantly, but the pH sensitivity of the dye is preserved. Figure 7A shows representative FLIM images of cheese matrix buffered to pH 3.0–6.0 and stained with Oregon Green 488. The pH sensitivity of the dye is immediately apparent from the change of the pseudocolor encoding local fluorescence lifetime. The mean lifetime τ*_m_* calculated as a mean value from several fields of view is plotted against the buffer pH in Figure 7B (solid symbols and a linear least-squares fit). The mean lifetime of the dye changes by more than 1 ns in the studied pH range.

**Figure 8:**
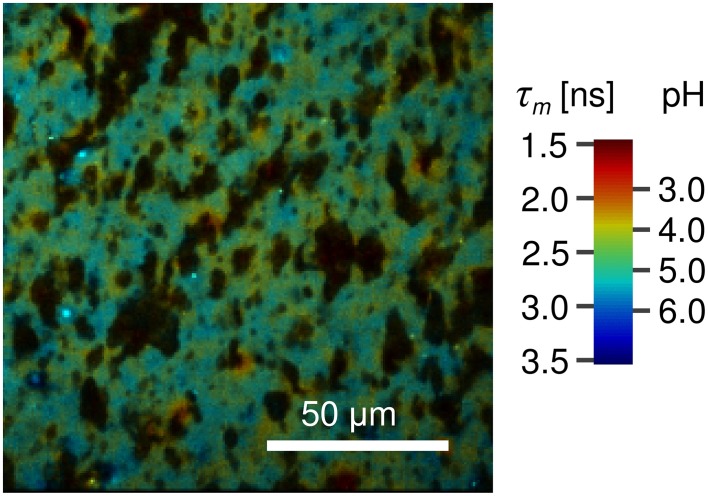
**FLIM image of natural cheese sample stained with Oregon Green 488**. Shown are apparent local variations of fluorescence lifetime and thus the pH. Localized spots with pH as low as 4.0 are observed. The dark areas most likely represent fat within the cheese matrix. The pseudocolor scale of the FLIM images is calibrated both in lifetime and pH values.

To measure the local pH of cheese the dye is applied to the cheese matrix without a pH buffer and the same procedure is followed. From the acquired FLIM image and the linear fit of the calibration series the average pH of vat 2 cheese sample after 223 days of ripening was determined to 5.2 ± 0.2 (Figure 7B, open symbol). The local variations of the fluorescence lifetime (and thus the pH) in a natural cheese sample are shown in greater detail in Figure 8. We note that application of the dye to the non-buffered cheese produced no measurable macroscopic pH change, as measured by a macroscopic pH electrode method.

## Discussion

We have confirmed that the C-SNARF-4 fluorescent pH probe may be used as a reliable ratiometric pH indicator at pH 5.5 and above (Figure 3). Essentially, the emission spectra do not depend on the excitation wavelength in the range 480–530 nm, as illustrated in Figure 2. However, the range of interest in cheese research is usually at lower pH values, generally around 4.8–6.0 (with some exceptions). In this range the traditional ratiometric approach does not provide sufficient sensitivity.

Excitation-emissions scans (Figure 4) show that the results of ratiometric pH measurements are largely insensitive to the excitation wavelength, but utilizing the lifetime information may extend the useful range of the dye toward lower pH's (Figure 5).

We have observed and measured the pH dependence of fluorescence lifetime of C-SNARF-4 dye, which extends through the pH range that can be sensed toward more acidic values (down to pH ≈ 3), part of this range which may be encountered in cheese research. From the calibration curve τ*_2_* in Figure 6B) one can clearly see the relationship between pH and fluorescence lifetime. But the calibrated lifetime–pH dependence performed in aqueous solutions is not directly applicable to cheese matrix results, as a new long-lifetime component appears τ_3_ in Table 3) and the lifetimes of the other two components are significantly shortened in such a protein-rich environment. More specifically, the τ_2_ component measured on cheese samples (about 1.7 ns, Table 3) would correspond to pH below 4.0 (according to the calibration curve τ_2_ in Figure 6B), which is much lower than the reference macroscopic value (about pH 5.0, Table 2). A similar interaction of SNARF-based dye with proteins was described previously (Srivastava and Krishnamoorthy, 1997). We also note that we observed strong affinity of the fluorescent probe toward the protein-rich cheese surface, which changes the dye's photophysical properties significantly and alter its pH response. Both the spectral and lifetime signatures of the stained protein matrix do not correspond to the calibration values at any pH.

Moreover, we observed that both the fluorescence intensity and lifetimes are essentially homogeneous across the sample surface (as shown by the small standard deviations in Table 3). The expected pH variation between samples is less than 0.5 and cannot be reliably detected by the C-SNARF-4 dye.

Similarly, we also applied the same methodology to other pH sensitive fluorescent dyes, namely BCECF-AM, FITC, and Acridine Orange, but again we observed little to no lifetime-pH sensitivity when applied to cheese matrix (data not shown).

On the other hand, our results suggest that applying the FLIM microscopic technique to the Oregon Green 488 fluorescent dye resulted in an accurate method of determination of pH in the cheese samples (the main results are summarized in Figure 7). The average value of pH 5.2 ± 0.2 is in good agreement with the macroscopic pH 5.3 (Table 2). In addition micro-heterogeneity in pH is apparent in the cheese matrix as show in Figure 8 with pH ranging between 4.0 and 5.5 depending on location. This is particularly interesting as it shows the pH of cheese matrix is not homogenous but contains localized variation of pH. This may be due to localized differences in the aqueous phase or concentrations of constituents of the aqueous phase including lactose, lactate, minerals or salt. It may also be influenced by variations in buffering capacity of the surrounding cheese matrix. It also poses questions regarding the influence of microbial colonies on pH micro heterogeneity. Other authors (Lopez et al., 2006, 2007; Pereira et al., 2009) reported bacterial colonies to be located at the protein fat interface, with the bacterial cells always being in contact with the fat globule membrane. Although the study of (Jeanson et al., 2013) showed no differences in pH around microbial colonies, that study differed in that it was conducted on an unripened non-fat UF model cheese system rather than on a ripened natural cheese matrix, and the microbial colonies were lactococci rather than thermophillic species.

The current study has focused on applying the developed method to determining the micro heterogeneity within one of the cheese types (vat 2). It is envisaged that future work would focus on using the developed method to determine whether manufacture processes influence pH at local level within different cheese matrices and whether different cheese types may have different patterns of micro heterogeneity. We also expect that this methodology can be employed to examine also wide range of different types of food products with sufficient moisture content.

### Conflict of interest statement

The authors declare that the research was conducted in the absence of any commercial or financial relationships that could be construed as a potential conflict of interest.
